# Spatial Distribution of *Taenia solium* Porcine Cysticercosis within a Rural Area of Mexico

**DOI:** 10.1371/journal.pntd.0000284

**Published:** 2008-09-03

**Authors:** Julio Morales, José Juan Martínez, Marcos Rosetti, Agnes Fleury, Victor Maza, Marisela Hernandez, Nelly Villalobos, Gladis Fragoso, Aline S. de Aluja, Carlos Larralde, Edda Sciutto

**Affiliations:** 1 Facultad de Medicina Veterinaria y Zootecnia, Universidad Nacional Autónoma de México, Ciudad de México, México; 2 Department of Informatics, University of Sussex, United Kingdom; 3 Instituto Nacional de Neurología y Neurocirugía, Ciudad de México, México; 4 Secretaría de Desarrollo Agropecuario, Dirección General de Ganadería, Gobierno del Estado de Morelos, Cuernavaca, México; 5 Instituto de Investigaciones Biomédicas, Universidad Nacional Autónoma de México, Ciudad de México, México; University of California Berkeley, United States of America

## Abstract

Cysticercosis is caused by *Taenia solium*, a parasitic disease that affects humans and rurally bred pigs in developing countries. The cysticercus may localize in the central nervous system of the human, causing neurocysticercosis, the most severe and frequent form of the disease. There appears to be an association between the prevalence of porcine cysticercosis and domestic pigs that wander freely and have access to human feces. In order to assess whether the risk of cysticercosis infection is clustered or widely dispersed in a limited rural area, a spatial analysis of rural porcine cysticercosis was applied to 13 villages of the Sierra de Huautla in Central Mexico. Clustering of cases in specific households would indicate tapeworm carriers in the vicinity, whereas their dispersal would suggest that the ambulatory habits of both humans and pigs contribute to the spread of cysticercosis. A total of 562 pigs were included in this study (August–December 2003). A global positioning system was employed in order to plot the geographic distribution of both cysticercotic pigs and risk factors for infection within the villages. Prevalence of pig tongue cysticercosis varied significantly in sampled villages (*p* = 0.003), ranging from 0% to 33.3% and averaging 13.3%. Pigs were clustered in households, but no differences in the clustering of cysticercotic and healthy pigs were found. In contrast, the presence of pigs roaming freely and drinking stagnant water correlated significantly with porcine cysticercosis (*p* = 0.07), as did the absence of latrines (*p* = 0.0008). High prevalence of porcine cysticercosis proves that transmission is still quite common in rural Mexico. The lack of significant differentiation in the geographical clustering of healthy and cysticercotic pigs weakens the argument that focal factors (e.g., household location of putative tapeworm carriers) play an important role in increasing the risk of cysticercosis transmission in pigs. Instead, it would appear that other wide-ranging biological, physical, and cultural factors determine the geographic spread of the disease. Extensive geographic dispersal of the risk of cysticercosis makes it imperative that control measures be applied indiscriminately to all pigs and humans living in this endemic area.

## Author Summary

*Taenia solium* cysticercosis is a parasitic disease that severely affects human health in underdeveloped countries and has re-emerged in North America. The adult parasite lives in the intestines of humans, where it thrives and sheds packages (proglottids) loaded with thousands of eggs that are, in turn, expelled upon defecation. Cysticercosis occurs after tapeworm eggs are ingested by an intermediate host (pig or human) and then hatch, migrate, and lodge in the host's tissues, where they develop onto larval cysticerci. Deficient hygiene, inadequate feces disposal, outdoor defecation, freely roaming pigs, and inadequate meat inspection promote transmission. Success in lowering transmission is limited by the complex network of biological and social factors that maintain the endemia. Effective control will require social development and powerful, sustained interventions targeted at the transmission cycle's crucial nodes. Rural pigs are obligate intermediate hosts and thus prime targets for control through vaccination and treatment. It has been proposed that pigs be used as sentinels to monitor environmental *T. solium* contamination. We conducted a spatial study of cysticercotic pigs among 13 neighboring villages in rural Central Mexico, constituting the first step of an attempt to discover a cost-effective and accurately targeted control intervention. Cysticercotic pigs were not found clustered in specific households, suggesting that the risk of infection is widely dispersed in the area and indicating that extensive and inclusive control measures are needed.

## Introduction

Cysticercosis is caused by *Taenia solium*, a parasitic disease that affects humans and freely roaming pigs in developing countries [1]. In Mexico, cysticercosis persists because conditions ideal for the parasite's life cycle remain prevalent in many rural areas [2]. The most severe manifestation of the disease occurs when the larval phase of the parasite (cysticerci) lodge in the human central nervous system (CNS), causing neurocysticercosis (NC). NC is a common parasitic disease affecting the CNS and is still responsible for high morbidity and mortality rates in endemic countries [3],[4].

It has long been recognized that porcine cysticercosis is closely associated with the presence of freely roaming pigs that have access to sites contaminated with human feces [5]–[10]. Because of the prevalence of this form of pig husbandry in endemic countries, combined with problems associated with poverty and poor education, eradication of *Taenia solium* [11],[12] may appear to be unachievable in the near future. However, a number of traditional measures have proved effective in lowering levels of transmission in Mexican villages, including education, improvement of household sanitation, changes in meat inspection methods and the corresponding destruction of infected meat, treatment of tapeworm carriers, and modifications in pig-rearing methods [5],[13]. Additional control measures have also been devised: vaccines [14], more-effective treatment protocols [15], and improved methods for diagnosing taeniasis [16] and cysticercosis [17]. Nonetheless, huge costs and logistic complexity make most of these measures impractical for large-scale, long-term nationwide campaigns in developing countries.

Mexico's demographic and geocultural vastness poses additional challenges for nationwide control programs. Thus regional approaches are also recommended, especially as epidemiological studies point to variations in rural rearing methods, in different regions and villages [18]. Understanding the details of porcine cysticercosis microepidemiology in defined and limited areas may help to improve the efficacy of programs for controlling transmission. The short life span (about a year) of pigs reared in primitive conditions, the relatively restricted movements of even freely roaming pigs compared to humans, and the relative ease of diagnosing porcine cysticercosis in vivo, make pigs useful indicators of ongoing active sites of cysticercosis transmission. Sentinel hosts reared on quasiexperimental farms [19] were originally employed to study the dynamics of *Taenia ovis* transmission in ovine cysticercosis. These farms were later proposed as bioindicators of environmental contamination with *T. solium* eggs [20].

In the present study, a spatial analysis of rural porcine cysticercosis was conducted to determine whether the risk of infection is focused in an area or is widely dispersed, and subsequently whether massive or focal measures of parasite control are required.

## Materials and Methods

In 2003, a study of the prevalence of porcine cysticercosis (PC) in the 33 municipalities of the State of Morelos detected many villages with endemic PC in the “Sierra de Huautla” region [9]. This region includes 145,861 acres of tropical forest and a total of 50,000 inhabitants [21]. It is located in the southern part of the state of Morelos, between the geographical coordinates of 18°20′ and 18°31′ north latitude and 98°51′ and 98°53′ west longitude, within an area classified as a Biosphere Reserve and recently declared a Natural World Heritage site. The rainy season lasts from mid-June and to October/November, and the dry season lasts from January and to May.

**Figure 1:**
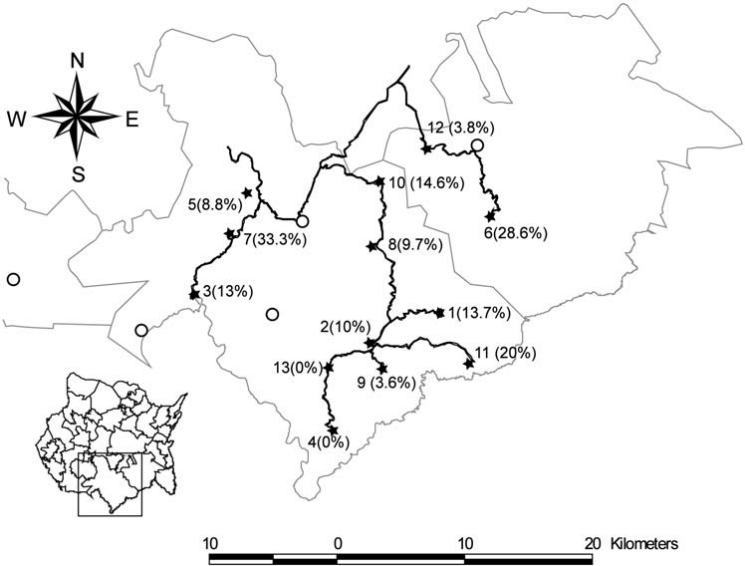
In the inferior left corner, a map illustrates the municipalities of the Mexican state of Morelos and the localization of the Sierra de Huautla. The main figure shows a map of the “Sierra de Huautla” displaying the studied villages marked by stars as well as their prevalence. Other villages in the region, marked by open circles, and main roads are shown. The distance bar applies to the largest map.

The present study was carried out in 13 of the 25 villages located in the Sierra de Huautla. The 13 villages included are located in the road-accessible area of the eastern slope of Sierra de Hautla and represent 72% (13/18) of all villages in the area (Figure 1). The criteria for selecting villages included a high prevalence of roaming pigs and the existence of previous individual reports indicating the presence of *T. solium* porcine cysticercosis (J. Morales, personal observations). The villages share cultural, commercial, social, and economic characteristics. Health facilities are scarce, drainage is lacking, and only the main streets are paved. Most villages have electricity (90%) and rural telephone services (70%). The public water supply system is dependent on wells and water deposits (80%) and water is not potable.

Pig phenotypes in the area are extremely diverse. The animals mate casually and 85% roam freely in streets, backyards, and nearby pastures, frequently finding and ingesting human feces that can be contaminated with parasite eggs. During the rainy season, a number of pigs (15%) are tethered in improvised pens to prevent them from damaging areas of farm production. Some pigs (8%) are occasionally fed with commercial concentrates and corn. Most of the time pigs drink residual water from ravines, dikes, and borders and that is sometimes contaminated with human feces. Animals in some villages are provided tap water from the public system (21%) and from wells (17%) when water is scarce. The remaining free-ranging pigs drink stagnant water that is often accumulated from household drainage run-off. Almost all (96%) of the village pigs were born in the community, 2% were introduced from other municipalities, and the remainder were introduced from other federal entities. The predominant pig phenotype is “native” (58%), and 42% are descended from “mixed” breeds (York, Landrace, Duroc, Hampshire, and Pietrain). Twenty percent of pigs are slaughtered and consumed by their owners, 13% are sold to butchers in the village, and the remaining 67% are sold at minimal prices to pig traffickers and later resold outside the area, in neighboring communities and cities. Most male pigs are castrated a few months prior to being slaughtered.

### Diagnosis of pig cysticercosis

The present study was carried out between August and December 2003. A total of 562 free-roaming pigs over 3 mo of age (61% of the pigs in the area [V. Maza personal communication]), regardless of sex, were included in this study and their owner's permission was obtained for inspection by our team. Once the pig was caught and restrained, its tongue was examined visually and in vivo palpation [6] was carried out to identify any subepithelial cysticerci. Six highly trained animal-health technicians supervised by a research team veterinarian confirmed diagnoses. In vivo PC diagnosis by tongue inspection manifests certain shortcomings: inspectors may vary in opinion as cysticerci not evident on the tongue may evade identification, and conversely lumpy lesions on the tongue may be wrongly identified as cysticerci. Our accuracy scores were 74% (17/23) sensitivity using tongue diagnosis, and 94% (8/206) specificity, when compared to the gold standard of necropsy diagnosis among rural pigs. Positive serology by antibody detection reveals previous exposure, not necessarily infection, whereas testing for circulating antigens is more accurate for diagnosing infection, but identifies only heavily infected animals [22],[23]. Furthermore, the strong resistance of pig owners to permitting more invasive forms of diagnosis, for example those that require bleeding, biopsy, or premature slaughter for autopsy, make tongue diagnosis the only practical way of examining hundreds of rural pigs in Mexico.

### Biological factors

The age (months), weight (kg), and phenotype (native or mixed breed [York, Landrace, Duroc, Hampshire, and Pietrain]) were recorded for each pig. Veterinarians also determined whether sows were pregnant at the time of inspection and whether boars had been castrated, and if so, at what age. As pregnancy was detected only by physical inspection, early pregnancies probably passed unnoticed.

### Exposure factors

A questionnaire was administered to the pigs' owners in order to obtain information about how pigs were kept (i.e., whether drinking water came from; a well, a river, or a tap; whether the pig was allowed to roam freely at all times or was occasionally tethered). Additionally, owners' households were inspected to determine whether a latrine existed.

### Geo-referenced location of pig-rearing households

**Figure 2:**
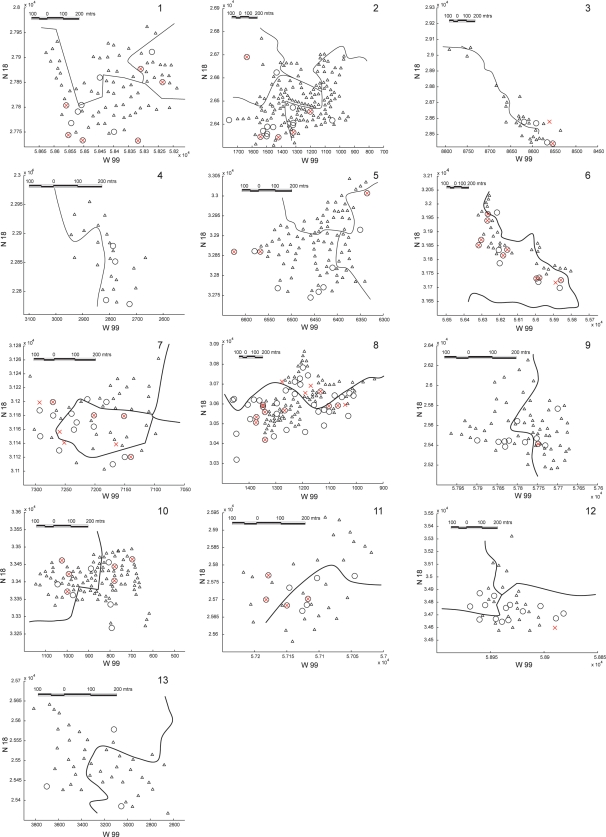
The geo-referenced location of the households with non-cysticercotic (O), cysticercotic (×), and both cysticercotic and non-cysticercotic pigs (⊗) with a schematic representation of the position of households (Δ) in each of 13 villages in the study.

The location of households that contained pigs was recorded using a global positioning system (Garmin Etrex Venture1). The actual location recorded was that of the corrals adjacent to the house. Using ArcView 9.0 ArcGis (2006; ESRI, http://www.esri.com/) the geo-referenced location was placed on orthomaps kindly provided by Valentino Sorani, LISIG-UAEM. Schematics of the orthomaps were used to represent the distribution of houses in the village and mark the position of the pig rearing households included in the study, illustrated in Figure 2.

### Clustering

The spatial position of each household was identified with a hand-held GPS, which established the paired coordinates. The location and numbers of healthy and cysticercotic pigs residing at each of these household positions were recorded. The coordinates for each village were normalized separately (applying values from 0 to 1) to eliminate the potential effects of units of scale and distance and thus permitting specific comparison for some of the indices used [24]. Three different indices were calculated for all pig-rearing households in each village, for those containing cysticercotic pigs, and for those with healthy pigs.

First, a quadrant analysis was applied, which requires laying a grid of equally sized quadrants over a map showing distribution; then the number of households in each quadrant were counted, with *n_i_* representing the number of households in each quadrant *i* [24]. The variance-to-mean ratio (VMR) was used to evaluate the level of dispersion of the households, expressed as: variance of *n_i_* over mean of *n_i_*. To account for the variation in the number of pigs in each household a second index, VMR_2_, was calculated, using the number of pigs in the households included in each quadrant. Thus, *p_i_* is the number of pigs in the quadrant/total number of pigs in the village and VMR_2_ is the variance of *p_i_* over the mean of *p_i_*.

The nearest neighbor index (NNI) employs the distance between occupied quadrants in order to evaluate the organization of the spatial distribution of the clusters [24],[25]. The NNI is calculated by the division
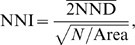
where *N* is the number of occupied quadrants and 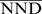 is the mean of the distance from every point to its nearest neighbor. The interpretation of the index values is presented in Table 1. The grid resolution for all the indices is chosen, having once evaluated the different sizes and chosen the larger size, so that the values of the indices remain visually similar. In this case, the same grid was used for every town, consisting of 25 quadrants of side length of 0.20 each.

**Table 1:** Interpretation values for cluster indices.

Distribution	Indices		
	VMR	VMR2	NNI
Uniform	0	0	2.14
Random	∼1	∼1	∼1
Clustered	>1	>1	∼0

### Statistical analysis

Recorded data were stored in a database using Excel (Microsoft 2000). Data were statistically analyzed using SPSS10, InStat3, and GraphPad Software(1992–1998 GraphPad Software, http://www.graphpad.com). Qualitative results were compared, using a Chi-square test or a Fisher's exact test, in order to measure statistical significance. Prevalence odds ratios and their 95% confidence intervals (CIs) were employed to assess the strength of the association. A multivariate analysis was applied in order to assess risk factors by using logistic regression and retaining all the factors for which the *p*-value was ≤0.25. The threshold for significance was set at 0.05 for all statistical analyses.

### Ethical approval

The study protocol was approved by the ethics committee of the Instituto de Investigaciones Biomédicas, Universidad Nacional Autónoma de México. No experiments were performed on either humans or animals. The procedures reported herein were: (1) in humans resident in the rural villages, the application of questionnaires concerned with their home facilities and lifestyles, as well as with their forms of pig-raising; and (2) in pigs, the routine visual and manual exploration of their tongues by a professional veterinary, conducted according to the principles set forth by the Mexican Ethical Committee for the Care and Use of Farm Animals. Pig owners gave their written informed consent for the publication of questionnaire results and permission to examine the tongues of their pigs.

## Results

### Prevalence of porcine cysticercosis

Tongue cysticercosis was detected among 13.3% [95% CI, 13.27%–13.33%] of the 562 pigs (31% boars and 69% sows) with a statistically significant variation (*p* = 0.002) between villages of 0%–33.3% (Table 2). No relationship was evident in terms of between-village distances or with the total number of resident pigs (*p*>0.05).

**Table 2:** Prevalence of pig cysticercosis in 13 rural villages of the Sierra de Huautla, Morelos.

Village (Number Code)	Total Number^a^	Number of Inspected Pigs (%)	Number of Positive Pigs	Prevalence [95% CI]
Ajuchitlan (1)	129	73 (56)	10	13.7 [13.6–13.78]
Huautla (2)	175	60 (34)	6	10.0 [9.9–10.1]
Huixaztla (3)	37	23 (62)	3	13.0 [12.85–13.15]
Huaxtla (4)	30	16 (53)	0	0
La Era (5)	50	34 (68)	3	8.8 [8.67–8.9]
El Limón (6)	70	56 (80)	16	28.6 [28.48–28.72]
Los Elotes (7)	35	33 (94)	11	33.3 [33.1–33.47]
Quilamula (8)	160	134 (84)	13	9.7 [9.65–9.75]
Rancho Viejo (9)	50	28 (56)	1	3.6 [3.53–3.67]
San Jose de Pala (10)	70	41 (58)	6	14.6 [14.48–14.71]
Santiopan (11)	50	25 (50)	5	20.0 [19.83–20.17]
Tepehuaje (12)	40	26 (65)	1	3.8 [3.72–3.88]
Xochipala (13)	30	13 (43)	0	0
Total	926	562 (61)	75	13.3 [13.27–13.33]

a The number of pigs was estimated based on unpublished records available as of 2004 at the Secretaría de Desarrollo Agropecuario, Gobierno del Estado de Morelos, Departamento de Sanidad Animal, Cuernavaca Morelos.

### Relevant exposure risk factors

Table 3 presents a list of village features and household habits found to correlate significantly with PC: i.e., lack of latrines (*p* = 0.0008, OR = 2.4); allowing pigs to roam freely (*p* = 0.07, OR = 2.2) or to drink stagnant rainwater (*p* = 0.07, OR = 1.6). Regarding biological factors, early castration of males (4 mo before slaughter) significantly increased cysticercosis prevalence (*p* = 0.001, OR = 17.3).

**Table 3:** Risk factors related to cysticercosis in the 13 rural villages of the Sierra de Huautla, Morelos.

Variable	With Cysticerci	Without Cysticerci	*p*-Value	Crude Odds Ratios [95% CI]
Latrine (Y/N)	36/39	334/153	0.0008	2.4 [1.45–3.87]
Free ranging (Y/N)	69/6	410/77	0.07	2.2 [0.9–5.1]
Water supply: stagnant water from rain (Y/N)	54/21	298/189	0.07	1.6 [0.95–2.79]
Sex (M/F)	29/46	191/296	0.93	1.02 [0.6–1.69]
Male castration (Y/N)	21/8	139/52	0.97	1.02 [0.42–2.44]
Males castrated between 5 and 12 mo of age (>4 mo^a^/≤4 mo)	9/1	40 / 77	0.001	17.3 [2.1–141.6]
Gestating females (Y/N)	10/36	74/222	0.63	0.83 [0.39–1.76]
Phenotype (native/cross-breed)	42/33	284/203	0.7	0.91 [0.56–1.48]

a Pigs castrated at least 4 mo before slaughter.

Absence of latrines (OR = 13.3 [2.4–72.7]; *p* = 0.003) and early castration (OR = 24.76 [2.8–217.4]; *p* = 0.004) both represented factors closely linked with cysticercosis, as determined by binary logistic regression.

### Spatial location and prevalence

The clustering indexes show that the spatial distribution of pig-rearing households ranged from random (0.83–1.06) to clustered (1.24–2.33) according to VMR, and clustered (<0.29) for NNI (see Table 4). The households that had healthy pigs, which were in the majority, had a distribution similar to the total of households; both had nearly equal clustering indices. For the households with infected pigs, only villages 1, 5, and 6 manifested clustering (1.14, 1.61, and 1.46, respectively) and this was therefore very similar to the clustering indices recorded in all of the households. It is particularly evident (Figure 2) that the distribution of the infected pigs in these villages was very similar to the distribution of households and was therefore not related to the distribution of infection. Every case in Table 4 shows the NNI for the distribution of households with infected pigs to be higher, indicating less clustering, than that observed for the households with healthy pigs and for households overall.

**Table 4:** Clustering indices for all pig-rearing households (A), those with healthy pigs (H), and those with cysticercotic (C) pigs.

Village	*VMR* a			*VMR_2_* b			*NNI* c		
	A	H	C	A	H	C	A	H	C
1	1.24	1.24	1.14	9.39	8.57	2.29	0.17	0.17	0.22
2	2.33	2.33	0.83	9.62	8.94	1.14	0.18	0.18	0.22
3	1.25	1.40	0.96	10.41	9.27	1.61	0.25	0.29	0.35
4	0.87	0.87	—	3.63	3.63	—	0.25	0.25	—
5	1.67	1.67	1.61	6.30	6.07	1.61	0.20	0.20	0.35
6	1.81	1.65	1.46	7.26	4.95	2.98	0.18	0.18	0.20
7	1.06	0.95	0.97	2.69	1.99	1.34	0.13	0.15	0.20
8	1.57	1.49	0.98	10.26	10.03	0.98	0.12	0.12	0.16
9	1.91	1.91	—	7.32	7.25	—	0.19	0.19	—
10	0.89	0.89	0.79	3.70	3.10	0.79	0.16	0.16	0.20
11	0.83	0.83	0.87	2.83	3.02	1.25	0.17	0.17	0.25
12	1.25	1.20	—	4.21	4.33	—	0.16	0.16	—
13	0.92	0.92	—	5.47	5.47	—	0.29	0.29	—

Missing values show villages in which there were fewer than two households containing cysticercotic pigs.

a Variance to mean ratio.

b Variance to mean ratio using the frequency of pigs per household.

c Nearest neighbor index.

The values for the VMR_2_ show a similar pattern, in which frequencies of pigs in every household produced a large clustering value for the distribution of total households and for those containing only healthy pigs (>2.69 and >1.99, respectively). Nonetheless, the values for those containing pigs infected with cysticercosis were less in all cases, due to the relatively small number of infected pigs, when compared to the greater numbers of healthy animals; therefore there was less clustering among infected pigs.

A similar lack of clustering of cysticercotic cases was observed when examining only pigs younger than 5 mo, a subsample most likely to detect focal points of transmission presumably on account of their more sedentary behavior as compared to adult pigs. Finally, no clustering indices for cysticercotic pigs were revealed for 30% of the villages, since villages 4 and 13 presented no cases of cysticercotic pigs and villages 9 and 12 showed very low prevalence (3%).

## Discussion

This epidemiological study identified 13.3% (75/562) of the pigs in 13 villages of the ecological reserve Sierra de Huautla as suffering from tongue cysticercosis, with significant variations existing between villages (0%–33.3%). The significant differences in prevalence between communities did not appear to relate either to the measured risk factors or to the total number of pigs in the village.

The high levels of prevalence recorded in this study dispel optimistic hopes of cysticercosis having been eradicated in Mexico [26] and emphasize the importance of applying measures to control transmission in rural areas of central Mexico. Our findings concur with those from previous studies [5]–[9],[13], confirming the importance of preventative measures, such as the construction of latrines and the confinement of rural pigs, to reduce risk of infection. Likewise, the significant increase in cysticercosis infection that we recorded among castrated male pigs and pregnant sows corroborates previous observations [8],[9] and points to the role played by sex steroids in murine cysticercosis, as described in more detailed studies [27].

For pigs under 5 mo of age, 21% (16/75) demonstrated less risk of cysticercotic infection compared to older pigs (*p*<0.0001), possibly either due to their more sedentary behavior or because they were disadvantaged in the competition to forage when roaming free [10], or because of passive transfer of maternal immunity [28].

The use of spatial information to evaluate clustering of PC is a novel approach that attempts to detect the most active sites for PC transmission in an endemic area. This type of analysis has contributed to epidemiological studies of other diseases (i.e., rabies, bovine tuberculosis, schistosomiasis [29]–[32]). This study indicates that the risk of cysticercosis is widely dispersed throughout rural pig-rearing households rather than being focused in specific households. Consequently, it is unlikely that sentinel pigs will be useful in identifying tapeworm carriers. Thus, for the rural pigs of this region, the infection risk of living close to a putative tapeworm carrier would appear to be lower than that of roaming free in search of food and ingesting contaminated human excrement. The roaming behavior of pigs that cover large distances (2–5 km) daily [10] may partially account for the dispersed distribution of PC in the area. In addition, the perambulation of rural workers while in the fields, tending to crops and herds, or waiting on the road for transportation provides the occasional tapeworm carrier with ample opportunities to disperse parasite eggs on soil and agricultural produce. The combination of the mobile patterns of both pigs and humans in this endemic area seems to be a cause for many of the PC cases in the Sierra de Huautla.

Our findings differ from other studies that report aggregation of cysticercotic pigs in specific households within villages is an important factor in transmission [7],[13],[33]. Besides regional, climatic, and cultural differences that may influence the geographic distribution of the risk of PC, discrepancies between our study and the other reports may also be due to our distinguishing the distribution of cysticercotic pigs from that of all pigs. Specific clustering of the infected pigs must significantly differ from that of all pigs for a claim of clustering of the risk of transmission to be supported.

No significant differences in particular risk factors were found between villages, although PC prevalence values varied greatly. It was to be expected that cysticercotic pig clusters would indicate the presence of a tapeworm carrier in the vicinity of certain villages, conforming with a recent report claiming that seroprevalence increases with decreasing distances to a tapeworm carrier [34]. However, this was not evident from our research in the Sierra de Huautla rural area. Instead, the distribution of cysticercotic pigs implied such a dispersed risk of infection that human factors emerged as more important determinants of parasite transmission (e.g., internal migration, family ties, human lifestyles, pig-rearing practices).

The findings presented here may not be representative of all rural regions of Mexico, as variations may exist relating to the local ethnic population and economic, demographic, cultural, and geographic conditions affecting routes of cysticercosis transmission. However, if we discard the premise that PC has a focal distribution, and instead show—as we have here—that risk factors in a given region are geographically broadly dispersed, then it follows that applying inclusive control measures at a region-wide level, rather than implementing localized or individually focused methods, will be the most effective approach to controlling cysticercosis.
